# Neurological Instability in Ischemic Stroke: Relation with Outcome, Latency Time, and Molecular Markers

**DOI:** 10.1007/s12975-021-00924-2

**Published:** 2021-06-24

**Authors:** Ramón Iglesias-Rey, Andres da Silva-Candal, Manuel Rodríguez-Yáñez, Ana Estany-Gestal, Uxía Regueiro, Elena Maqueda, Paulo Ávila-Gómez, José Manuel Pumar, José Castillo, Tomás Sobrino, Francisco Campos, Pablo Hervella

**Affiliations:** 1 Clinical Neurosciences Research Laboratory, Health Research Institute of Santiago de Compostela (IDIS), 15706 Santiago de Compostela, Spain; 2 Stroke Unit, Department of Neurology, Hospital Clínico Universitario, Santiago de Compostela, Spain; 3 Unit of Methodology of the Research, Health Research Institute of Santiago de Compostela (IDIS), Santiago de Compostela, Spain; 4 Department of Neuroradiology, Hospital Clínico Universitario, Santiago de Compostela, Spain

**Keywords:** Stroke, Neurological instability, Latency time, Glutamate, Interleukin

## Abstract

The National Institutes of Health Stroke Scale (NIHSS) is commonly used to evaluate stroke neurological deficits and to predict the patient’s outcome. Neurological instability (NI), defined as the variation of the NIHSS in the first 48 h, is a simple clinical metric that reflects dynamic changes in the area of the brain affected by the ischemia. We hypothesize that NI may represent areas of cerebral instability known as penumbra, which could expand or reduce brain injury and its associated neurological sequels. In this work, our aim was to analyze the association of NI with the functional outcome at 3 months and to study clinical biomarkers associated to NI as surrogate biomarkers of ischemic and inflammatory penumbrae in ischemic stroke (IS) patients. We included 663 IS patients in a retrospective observational study. Neutral NI was defined as a variation in the NI scale between − 5 and 5% (37.1%). Positive NI is attributed to patients with an improvement of > 5% NI after 48 h (48.9%), while negative NI is assigned to patients values lower than − 5% (14.0%). Poor outcome was assigned to patients with mRS ≥ 3 at 3 months. We observed an inverse association of poor outcome with positive NI (OR, 0.35; 95%CI, 0.18–0.67; p = 0.002) and a direct association with negative NI (OR, 6.30; 95%CI, 2.12–18.65; p = 0.001). Negative NI showed a higher association with poor outcome than most clinical markers. Regarding good functional outcome, positive NI was the marker with the higher association (19.31; CI 95%, 9.03–41.28; p < 0.0001) and with the highest percentage of identified patients with good functional outcome (17.6%). Patients with negative NI have higher glutamate levels compared with patients with neutral and positive NI (p < 0.0001). IL6 levels are significantly lower in patients with positive NI compared with neutral NI (p < 0.0001), while patients with negative NI showed the highest IL6 values (p < 0.0001). High glutamate levels were associated with negative NI at short latency times, decreasing at higher latency times. An opposite trend was observed for inflammation, and IL6 levels were similar in patients with positive and negative NI in the first 6 h and then higher in patients with negative NI. These results support NI as a prognosis factor in IS and the hypothesis of the existence of a delayed inflammatory penumbra, opening up the possibility of extending the therapeutic window for IS.

**Supplementary Information:**

The online version contains supplementary material available at 10.1007/s12975-021-00924-2.

## Introduction

The ischemic penumbra is the area of the brain surrounding the infarcted area that is potentially salvageable by an effective reperfusion during the first 24 h after an ischemic stroke (IS) [1, 2]. Recently, it has been suggested the existence of another penumbra, named inflammatory penumbra, caused by the inflammatory response around the infarcted area [3–5]. Unlike the ischemic penumbra, which is recruited by the ischemic core within the first 24 h after the stroke, the inflammatory penumbra is believed to last between 1 and 5 days [5] and could be treated by blocking the immune response [3, 4]. Both ischemic and inflammatory penumbrae are unstable regions of the brain that can evolve either into infarcted or normal brain tissue, with dramatic consequences in the clinical evolution of IS patients.

Clinical markers based on the National Institutes of Health Stroke Scale (NIHSS) are commonly used to evaluate stroke progression and to predict the patient’s outcome. Among those markers, early neurological deterioration (END), defined as an increase of 4 points or more in the NIHSS in the first 72 h, is frequently associated with increased risk of functional disability and mortality [6, 7]. Early dramatic recovery (EDR), defined as a decrease of 10 or more points in the NIHSS within the first 24 h or a decrease to a NIHSS score of ≤ 3 by the end of infusion, is relatively frequent in patients with middle cerebral artery stroke after intravenous recanalization [8]. Clinical–diffusion mismatch (CDM) defined as a NIHSS ≥ 8 and diffusion-weighted imaging lesion volume < 25 mL has been used as a marker of ischemic brain at risk of infarction and to recognize instable and potentially salvageable ischemic tissue, i.e., ischemic penumbra [9, 10]. The abovementioned clinical markers are useful selection tools to predict the success rate of reperfusion therapies; however, those markers were not studied in relation with the ischemic and inflammatory penumbra during the acute and sub-acute phases of IS.

In a previous study, we have demonstrated the benefits of using a dynamic neurological scale based on the static measurements of stroke severity measured by the NIHSS [11]. Neurological instability (NI), defined as the variation in the NIHSS in the first 24–48 h, may be a simple clinical metric that reflects dynamic changes in the area of the brain affected by the ischemia [12] and could be considered as an indicator of the balance between the repairing and damaging mechanisms occurring in the brain. Therefore, we hypothesize that NI could be associated with the evolution of the ischemic and inflammatory penumbra.

In this work, our aim is to test clinical markers associated with the evolution of the ischemic and inflammatory penumbra in IS patients. We analyzed the relation between NI and the clinical outcome of IS patients. Then, we analyzed the temporal profile of NI in relation with excitotoxicity (glutamate), as a measure of early damage in IS in relation with the ischemic penumbra, and with inflammation markers (interleukin 6, IL6) as a measure of late damage and inflammatory penumbra. We hypothesize that NI could be related with the existence of areas of cerebral instability, i.e., penumbra, that can expand or reduce the brain injury and the associated neurological sequels.

## Methods

### Study Design

The aim of this study was to evaluate NI as a clinical marker for IS evolution. First, we evaluated the relationship between NI and the clinical outcome at 3 months. Then, we studied the clinical factors influencing NI, and finally, we correlated the variations of glutamate and IL6 levels over time with NI.

This is a retrospective analysis conducted on a prospective registry of IS patients consecutively admitted in the stroke unit of the University Clinical Hospital of Santiago de Compostela (Spain) from January 2015 to March 2018. We prospectively included all IS patients admitted to the stroke unit in the BICHUS registry. The study was carried out in accordance with the Declaration of Helsinki of the World Medical Association and approved by the Research Ethics Committee of Santiago (2019/616). Written informed consent was obtained from each patient or from their relatives after full explanation of the procedures.

### Clinical Variables

Neurological instability was defined as NIHSS at admission — NIHSS at 48 h) × 100/42. This scale gives the percentage of variation in the NIHSS normalized to its maximum possible value, which is 42. Neutral NI was defined as a variation in the NI scale between − 5 and 5%. Positive NI is attributed to patients with an improvement of > 5% NI after 48 h, while negative NI is assigned to patients values lower than − 5%. The patients deceased in the first 48 h were assigned with a NI of − 100%.

Early neurological deterioration (END) was defined as an increase of ≥ 4 points in the NIHSS in the first 72 h. Early neurological improvement (ENI) was defined as a decrease of ≤ 8 points in the NIHSS in the first 24 h. Early dramatic improvement (EDI) was defined as a decrease of ≤ 10 points in the NIHSS in the first 24 h. Clinical diffusion mismatch was assigned to patients with a NIHSS ≥ 10 and a diffusion-weighted image (DWI) ≤ 25 mL. Latency time, or time to hospital arrival, was defined as the time between the onset of stoke symptoms and the hospital arrival. Hemorrhagic transformation type I was assigned by computed tomography based on the presence of a region of hyperdensity in the infarct zone. Any reperfusion treatment was assigned to patients treated either with tPA, mechanical thrombectomy, or a combination of both.

Lesion volumes were determined using the ABC/2 method until 2016 and through the automated planimetric method from then onwards. Infarct growth was calculated as follows: (Computed Tomography (CT) volume at 4^th^ − 7^th^ day − Diffusion Weighted Image (DWI) at admission) × 100/CT volume at 4^th^ − 7^th^ day.

Neurological deficits were determined by the NIHSS upon admission and every 6 h during the hospitalization in the stroke unit. For this study, the NIHSS was assessed at admission and after 48 h. Functional outcome was assessed by the modified Rankin scale (mRS) at discharge and at 3 months ± 15 days. Good outcome was assigned to patients with mRS ≤ 2 at 3 months.

Both scales were evaluated by internationally certified neurologists, supervised by the same neurologist.

### Patients

We included 663 IS patients in a retrospective observational study. The inclusion criteria were admitted to the stroke unit from January 2015 to March 2018; IS with known onset and < 12 h evolution confirmed by neuroimaging; previous modified Rankin scale ≤ 2; no comorbidity associated with life expectancy < 3 months; without known acute or chronic inflammatory process; not included in clinical trials; with DWI on admission; with NIHSS on admission, 24 and 48 h; follow-up for 3 months evaluated with modified Rankin scale at 3 months; and with serum sample collected at admission and stored at − 80 ºC.

### Analytical Methods

Biochemistry, hematology, and coagulation tests were assessed in the central laboratory of the hospital. For the molecular determinations, venous blood samples were collected in Vacutainer tubes (Becton Dickinson, San Jose, CA, USA) on admission and 24 ± 12 h and/or 48 ± 12 h from stroke onset. After clotting for 60 min, blood samples were centrifuged at 3000 × g for 10 min, and the serum was immediately aliquoted, frozen, and stored at − 80 ºC until analysis.

Glucose levels, glycosylated hemoglobin, leukocytes, red blood cells, platelets, fibrinogen, C-reactive protein, total and fractionated cholesterol, triglycerides, proBNP, vitamin D, and cholecalciferol were measured on admission. Serum glutamate levels were determined on admission by high-performance liquid chromatography (HPLC) analysis following a method described elsewhere [13]. Serum levels of IL6 were also measured at admission using an immunodiagnostic IMMULITE 1000 System (Diagnostic Products Corporation, CA, USA).

### Statistical Analysis

First, an analysis was performed describing the sample in regard with the clinical outcome at 3 months. Kolmogorov–Smirnov test with Lilliefors correction was applied to assess normality. Frequencies and percentages were calculated for categorical variables, while the continuous variables were expressed by mean values ± standard deviation (SD) or median values and interquartile range depending on their adjustment to normality. ANOVA and chi-squared tests were performed to determine differences in the variables between the groups (patients with good outcome vs patients with poor outcome). Latency times were categorized in 5 quintiles with an equal number of patients per group.

A first logistic regression analysis (unadjusted) was carried out using good outcome as a dependent variable for the following independent variables: hemorrhagic transformation type I, clinical diffusion mismatch, ENI, any reperfusion treatment, EDI, or positive NI. The same unadjusted logistic regression model was carried out using poor outcome as a dependent variable for the following independent variables: infarct volume, DWI volume at admission, CT volume at 4–7 days, NIHSS at admission, NIHSS at 48 h, negative NI, and END.

Logistic regression analyses were performed with the independent variables selected by their statistical significance in the bivariate analysis or by their clinical significance. These results were shown as odd ratios (ORs) with 95% confidence intervals (CI 95%). A p-value < 0.05 was considered to be statistically significant. Then, an analysis was carried out describing the sample in regard with the NI, categorized in neutral, positive, and negative, using the same methods described above. In order to evaluate the clinical factors influencing NI, a multivariate model was built using a linear regression analysis. The dependent variables were adjusted by clinically significant variables calculated in the multivariate analysis by ANOVA tests. Then, logistic regression analyses were carried out to evaluate the influence of latency time over negative NI. Here, the latency time was evaluated in 5 quintiles using the first quintile as reference. Finally, the same quintile groups were used to evaluate the levels of glutamate and IL6, using ANOVA tests to determine differences in the variables between the groups (neutral, positive, and negative NI).

All statistical analyses were conducted in SPSS 21.0 (IBM, USA).

## Results

### Association Between Functional Outcome and Neurological Instability

**Fig. 1:**
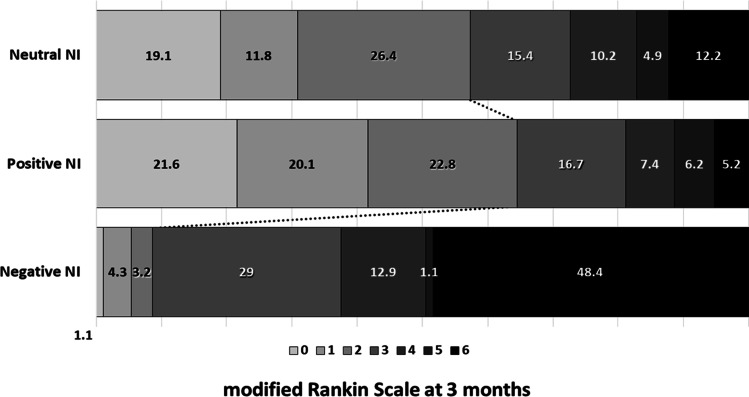
Three-month functional outcome (modified Rankin scale [mRS]) of ischemic stroke patients with neutral, positive, or negative neurological instability (NI)

We retrospectively included 663 IS patients in this study. The median average age was 73.0 ± 8.3, and 54.6% were males. The average NIHSS at admission and at 48 h were 15 ± 7 and 10 ± 8, respectively. Neurological instability (NI) was used to classify IS patients according to their NIHSS variation. NI was calculated from the variation between the NIHSS at admission and 48 h. Other time periods were also evaluated, and NI was also calculated between admission and 24 h and between 24 and 48 h (see Table S1). We observed that the results obtained from the variations between 0–48 h and 0–24 h were equivalent, while NIHSS variations between 24 and 48 h were not associated with the clinical outcome or IS patients. NI values showed a non-normal distribution (median 4.76 [0.0, 19.0]), and IS patients were categorized in three groups according to their variations of the NIHSS: neutral NI or no variation of the NIHSS, 246 patients (37.1%); positive NI or improvement in the NIHSS, 324 patients (48.9%); and negative NI or worsening in the NIHSS, 93 patients (14.0%). We observed an association between NI and the clinical outcome at 3 months, evaluated by the mRS (Fig. 1), being the positive NI group the one with the best outcome at 3 months, while the negative NI group showed the worst outcome. Among those patients with good outcome at 3 months (358), 58.4% had a positive NI, 39.4% presented neutral NI, and only a 2.2% showed a negative NI (p < 0.0001). On the other hand, poor outcome was observed in 305 patients, where 37.7% had positive NI, 34.4% had neutral NI, and 27.9% had a negative NI (p < 0.0001).

IS patients were classified regarding the functional outcome at 3 months (358 with good outcome and 305 with poor outcome), and significant differences were found for several clinical and analytical variables between the two groups (Table S2). A logistic regression analysis was then carried out including the clinically significant variables influencing the poor outcome at 3 months of IS patients (Table 1). The adjusted model showed an inverse association of positive NI with poor outcome (OR, 0.35; CI 95%, 0.18–0.67; p = 0.002) and a direct association with negative NI (OR, 6.30; CI 95%, 2.12–18.65; p = 0.001) in relation with neutral NI. The administration of any reperfusion treatment (thrombolysis, thrombectomy, or both) had a significant inverse association with poor outcome (OR, 0.21; CI 95%, 0.11–0.41; p < 0.0001).

**Table 1:** Logistic regression model for factors related with poor outcome at 3 months

	OR	CI 95%	*p*	OR*	CI 95%	*p*
Neurological instability						
Neutral	Ref	-	-	Ref	-	-
Positive	0.21	0.18–0.54	0.001	0.35	0.18–0.67	0.002
Negative	14.26	6.62–30.74	< 0.0001	6.30	2.12–18.65	0.001
Latency time	1.00	1.00–1.00	< 0.0001	1.00	1.00–1.00	0.001
Any reperfusion treatment	0.28	0.19–0.40	< 0.0001	0.21	0.11–0.41	< 0.0001
Glutamate at admission (uM)	1.01	1.00–1.01	< 0.0001	1.01	1.00–1.01	0.005
IL6 at admission (pg/mL)	0.998	0.98–1.02	0.841	0.93	0.89–0.96	< 0.0001

^*^Adjusted by age, sex, latency time, previous mRS, atrial fibrillation, temperature, NIHSS at admission, C-reactive protein, infarct growth, any reperfusion treatment, glutamate at admission and IL6 at admission, and neurological instability

The observed association between NI and functional outcome was compared with other clinical markers commonly used in the clinic (summarized in Fig. S1). Negative NI showed a higher association with poor outcome than most of the clinical markers. The calculated sensitivity of negative NI was as high as 0.978, despite having a low specificity of 0.28. Regarding the markers of good functional outcome, positive NI was the marker with the higher association (19.31; CI 95%, 9.03–41.28; p < 0.0001) and with the highest percentage of identified patients with good functional outcome (17.6%).

### Clinical Variables Associated with Neurological Instability

Once the association between NI and outcome at 3 months was demonstrated, we then analyzed the clinical factors influencing NI. The univariate analysis (Table S3) showed significant differences in the three NI groups (neutral, positive, and negative) in the following variables: previous mRS (p = 0.048); arterial hypertension (p = 0.003); atrial fibrillation (p = 0.033); time since transition ischemic attack less than 1 day (p = 0.007); C-reactive protein (p = 0.005); patients with any reperfusion treatment (p < 0.0001); latency time (p < 0.0001); axillary temperature at admission (p < 0.0001); and the subtype of ischemic stroke according to the TOAST classification (p < 0.0001).

A multivariate linear regression model was carried out adjusting by the clinical factors that showed statistical differences between groups (Table S4), and it was observed that latency time (β, − 0.03; CI 95%, − 0.045, − 0.023, p < 0.0001) and the temperature at admission (β, − 3.11; CI 95%, − 5.25, − 0.97; p = 0.005) have a negative association with NI, while reperfusion treatment has a positive association with NI (β, 5.93; CI 95%, 2.39, 9.45; p = 0.001).

**Fig. 2:**
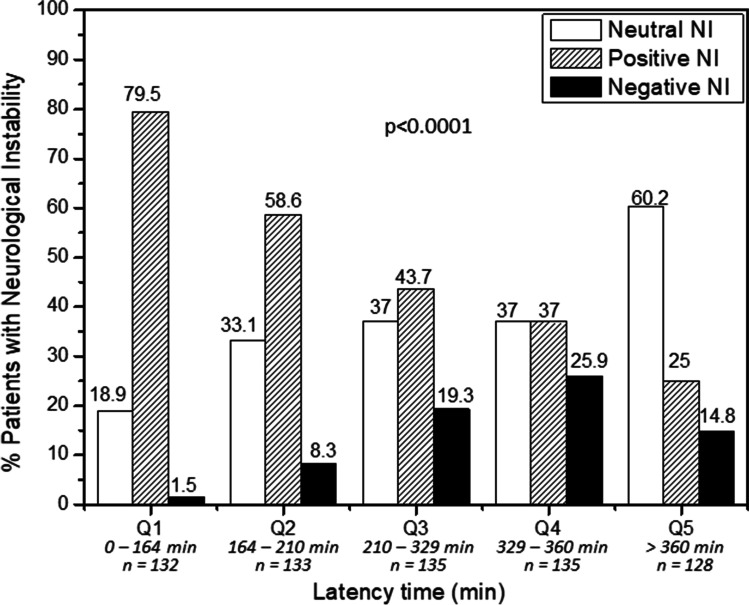
Percentages of patients with neutral, positive, and negative neurological instability (NI) in regard their latency time

In search for therapeutic opportunities, reperfusion treatment and latency time are the only factors associated to NI which have the possibility to be modified to improve the clinical outcome of IS patients. Therefore, in order to better study the association with NI, latency time was categorized in quintile (Q) groups (Fig. 2), and it was observed that positive NI was more frequent in patients arriving at the hospital in the first 210 min (Q1 and Q2), while neutral and negative NI were more frequent in patients arriving after 360 min (Q5, p < 0.0001). The percentage of patients with good outcome was already observed at NI measured between 0 and 24 h (Fig. S2), although the time window of 0–48 h showed a wider selection of patients with variation of the NIHSS. Also, we observed some small variations in the NIHSS in the 24–48-h period; therefore, the time range between 0 and 48 h represents better the changes in the NIHSS in IS patients.

A logistic regression model was carried out to analyze the probabilities of developing negative NI in regard the latency time and other factors (Table 2). We did not find significant differences between patients arriving in the first 164 min (Q1) and patients arriving between 164 and 210 min (Q2) (p = 0.459). Nevertheless, differences were found in IS patients arriving at the hospital 210–329 min (Q3) after the onset of symptoms, with 6.39 more possibilities to have negative NI than patients arriving in the first 164 min (p = 0.02). Patients arriving 329–360 min (Q4) increased the risk to develop negative NI to 15.34 (p < 0.0001). Interestingly, we did not find significant differences in the probability of developing negative NI in patients arriving after 360 min (Q5) in comparison with patients arriving in the first 164 min. Also, the differences found in the OR were independent of the presence of reperfusion treatments, although reperfusion influenced the probability of having negative NI (OR 0.41, p = 0.04).

**Table 2:** Logistic regression model for factors related with negative neurological improvement

	OR	CI 95%	*p*	OR*	CI 95%	*p*
Latency time						
Q1: 0–164 min	Ref			Ref		
Q2: 164–210 min	5.86	1.27–26.97	0.023	1.93	0.34–10.95	0.459
Q3: 210–329 min	15.51	3.60–66.80	< 0.0001	6.39	1.34–30.43	0.02
Q4: 329–360 min	22.75	5.34–96.85	< 0.0001	15.34	3.35–70.18	< 0.0001
Q5: > 360 min	11.33	2.58–49.73	0.001	3.71	0.72–19.19	0.117
Any reperfusion treatment	0.42	0.24–0.76	0.004	0.41	0.17–0.96	0.04

^*^Adjusted by previous mRS, arterial hypertension, atrial fibrillation, time since last TIA less than 1 day, axillary temperature ≥ 37.5, and C-reactive protein

### Molecular Markers Associated with Neurological Instability

Finally, the basal levels of glutamate and IL6 at admission were analyzed in relation with NI, as molecular markers of excitotoxicy and inflammation, respectively. The results showed that patients with negative NI have higher glutamate levels (125.0 ± 14.6 µM) compared with patients with neutral (46.4 ± 5.4 µM) and positive NI (53.3 ± 3.9 µM) (p < 0.0001). IL6 levels were significantly lower in patients with positive NI (4.3 ± 0.2 pg/mL) compared with neutral NI (8.3 ± 0.5 pg/mL), while patients with negative NI showed the highest IL6 values (11.8 ± 1.2 pg/mL) (p < 0.0001).

The multivariate linear regression analysis also showed an association between glutamate and IL6 levels with NI (Table S3) with a negative association observed for both glutamate and IL6 levels (β =  − 0.030; CI 95%, − 0.46, − 0.012; p < 0.0001; and β =  − 0.424, CI 95%, − 0.632, − 0.22; p < 0.0001, respectively).

**Fig. 3:**
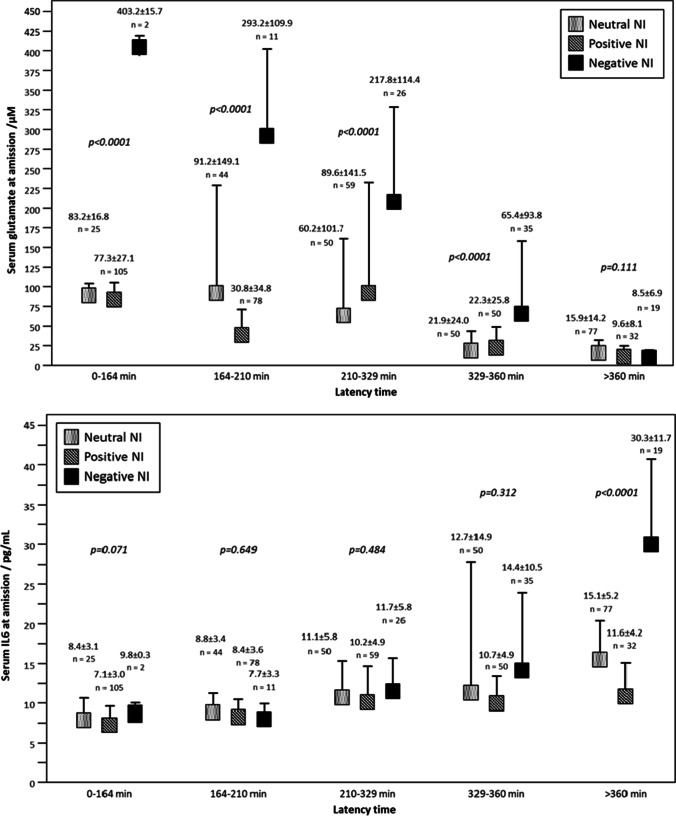
**a** Glutamate levels in ischemic stroke patients with neutral, positive, and negative neurological instability in regard the latency time. ANOVA tests were performed to evaluate differences in every latency time quintile. **b** IL6 levels in IS patients in with neutral, positive, and negative neurological instability in regard the latency time. ANOVA test was performed to evaluate differences in every latency time quintile

Glutamate and IL6 levels were also analyzed in regard with the latency time. It was observed that patients with neutral and positive NI have similar glutamate levels independent on the latency time. However, glutamate levels are much higher in patients with negative NI, and those levels decrease over time (Fig. 3). Conversely, IL6 levels increased over time for the three NI groups, with no differences found among them except for IL6 levels measured at latency times higher than 360 min. IL6 levels were significantly higher (p < 0.0001) for patients with negative NI arriving at the hospital 360 min after the stroke onset. The different profiles of glutamate and IL6 levels observed for patients categorized regarding their latency times could be attributed to the presence of an ischemic and inflammatory penumbra, as discussed next.

## Discussion

In this study, we proposed the use of NI, defined as the percentage variation of the NIHSS in the first 48 h, as a potent and simple tool to identify IS patients with active molecular mechanisms of either neuronal repair (positive NI) or deterioration (negative NI). In our study, we showed that NI is associated with the clinical outcome at 3 months. Patients with poor outcome at 3 months were predicted from negative NI with similar ORs to END predictions but with a higher percentage of identified patients. Good outcome was also predicted from positive NI, presenting an OR, and a number of patients identified much higher than other clinical markers such as clinical diffusion mismatch, ENI, or EDI. Taking all this into consideration, in our series, we observed that positive NI is the most potent marker of good outcome, while negative NI is the most efficient marker of poor outcome.

The association between the outcomes at 3 months with variations of the NIHSS between 0 and 48 h (NI) was not different to the variations between 0 and 24 h, suggesting that the molecular mechanisms taking place in the brain are still active after the first 24 h. In a previous work, we also evaluated the variations of the NIHSS between admission and hospital discharge, with an association with the clinical outcome at 3 months 6 times lower than the values predicted by NI [11].

NI was conditioned by several clinical factors, such as the functional situation of the patient before suffering the IS, hypertension, atrial fibrillation, hyperthermia, and levels of C-reactive protein; all of them are associated with negative NI and therefore with poor clinical outcome, as reported by other studies [14–16]. Positive NI, and consequently good outcome, was more commonly observed in patients who had a recent transient ischemic attack (TIA) and in those who have received any type of reperfusion treatment, demonstrating the association between recanalization and good outcome [16, 17].

Temperature, latency time, and reperfusion treatment are the only factors independently associated with negative NI that could be potentially addressed to improve the clinical outcome of IS patients. The association between temperature and good outcome is well-known [18–20], being hypothermia one of the most promising therapies in IS [21–23]. In addition, the association of latency time (time to hospital arrival) and the use of reperfusion therapies with the clinical outcome has been also demonstrated before [24]. Our findings using NI as clinical marker of IS are in good agreement with previous studies and encourage the management of temperature and the reduction of the latency time as much as possible, even beyond the time frame recommended for thrombolysis [25, 26].

Glutamate is an excitotoxic molecule that is released immediately after an IS, causing neuronal damage in the affected region [27, 28]. Therefore, reducing glutamate concentration is the aim of several experimental therapies [29–31]. Our findings showed that high glutamate levels are associated with poor outcome as observed in other cohorts of patients [32] and also with negative NI at short latency times, but this dependence is decreased over time. In fact, the influence of glutamate over NI was not observed in patients arriving at the hospital after 6 h, suggesting that the excitotoxic effect of glutamate is critical in the first 6 h, and consequently any treatment targeting glutamate excitotoxicity would have a maximum efficacy during that time frame (0–6 h). This observation is in agreement with early works on glutamate excitotoxicity, where a maximum glutamate concentration was reported at 6 h after stroke onset [33], and also with a recent publication where we have reported the association of glutamate with the late recovery of IS patients [11].

An opposite trend was observed for inflammation, where high basal IL6 levels were associated with good outcome, with a similar OR for logIL6 (0.35) to the values reported by Park et al. (0.45) [34]. It is well-known that IL6 levels tend to increase in the first 24 h, maintaining these levels up to 7 days [35]. Moreover, in our previous study, we observed that IL6 levels were higher 24 h after the IS onset in patients with no reperfusion treatment and in patients with an uneffective reperfusion, while patients subjected to an effective reperfusion treatment showed similar levels of IL6 on admission and at 24 h [11]. These observations are in agreement with the possible existence of a delayed inflammatory penumbra [3–5], maintaining high IL6 levels over time, which could be potentially used for extending the therapeutic window for IS.

## Conclusion

Neurological instability is a clinical marker that is related with the presence of active molecular mechanisms, either repairing or damaging the brain tissue. NI is associated with the clinical outcome at 3 months, and the molecular mechanisms associated to NI are related with the presence of an ischemic and inflammatory penumbra, although these relations must be confirmed with imaging studies. The presence of an inflammatory penumbra encourages the possibility of extending the therapeutic time window in acute ischemic stroke.

## Supplementary Information

Below is the link to the electronic supplementary material.

Supplementary file1 (DOCX 149 KB)
