# Supplementary material for: Neurological Instability in Ischemic Stroke: Relation with Outcome, Latency Time, and Molecular Markers
Source: Transl Stroke Res. 2021 Jun 24;13(2):228–37. doi: 10.1007/s12975-021-00924-2 (PMC8918467; doi:10.1007/s12975-021-00924-2)
Supplement: Supplementary file 1 — Supplementary file1 (DOCX 149 KB) [file 12975_2021_924_MOESM1_ESM.docx]

Supplementary Information

**Neurological Instability in Ischemic Stroke: relation with outcome, latency time and molecular markers.**

Ramón Iglesias-Rey PhD*^1^, Andres da Silva-Candal PhD^1^, Manuel Rodríguez-Yáñez MD PhD^2^, Ana Estany-Gestal PhD^3^, Uxia Regueiro PhD^1^, Elena Maqueda BSc^1^, Paulo Ávila-Gómez BSc^1^, José Manuel Pumar MD PhD^4^, José Castillo MD PhD^1^Tomás Sobrino PhD^1^, Francisco Campos PhD^1^, Pablo Hervella PhD*^1^

^1^Clinical Neurosciences Research Laboratory, Health Research Institute of Santiago de Compostela (IDIS), Santiago de Compostela, Spain.

^2^Stroke Unit, Department of Neurology, Hospital Clínico Universitario, Santiago de Compostela, Spain.

^3^Unit of Methodology of the Research, Health Research Institute of Santiago de Compostela (IDIS), Spain.

^4^ Department of Neuroradiology, Hospital Clínico Universitario, Santiago de Compostela, Spain.

*Corresponding authors

Dr. Pablo Hervella

Clinical Neurosciences Research Laboratory, Hospital Clínico Universitario, Travesa da Choupana s/n, 15706 Santiago de Compostela, Spain

Email: pablo.hervella.lorenzo@sergas.es

Phone: +34 981951086

Fax number: +34 981951098

Dr. Ramón Iglesias-Rey

Clinical Neurosciences Research Laboratory, Hospital Clínico Universitario, Travesa da Choupana s/n, 15706 Santiago de Compostela, Spain

Email: ramon.iglesias.rey@sergas.es

Phone: +34 981951086

Fax number: +34 981951098

**Table S1.** Comparison between the NI calculated from the variations in the NIHSS between 0-48h; 0-24h and 24-48 h. Adjusted by age, sex, latency time, previous mRS, atrial fibrillation, temperature, NIHSS at admission, C reactive protein, Infarct growth, any reperfusion treatment, glutamate at admission and IL6 at admission and neurological instability.

| **ΔNIHSS 0-48 h** | | | | **ΔNIHSS 0-24 h** | | | **ΔNIHSS 24-48 h** | | |
| --- | --- | --- | --- | --- | --- | --- | --- | --- | --- |
|  | **OR** | **CI 95%** | **p** | **OR** | **CI 95%** | **p** | **OR** | **CI 95%** | **p** |
| **NI** |  |  |  |  |  |  |  |  |  |
| **Neutral** | ref. | - | - | ref | - | - | ref. | - |  |
| **Positive** | 0.38 | 0.21-0.70 | 0.002 | 0.43 | 0.20-0.924 | 0.03 | 0.64 | 0.28-1.47 | 0.296 |
| **Negative** | 5.33 | 1.91-14.68 | 0.001 | 5.29 | 1.38-20.23 | 0.015 | 3.14 | 0.91-10.67 | 0.069 |

**Table S2.** Clinical and analytical variables by outcome groups (good and poor outcome) at three months.

| OUTCOME AT 3 MONTHS | | | |
| --- | --- | --- | --- |
|  | **Good** | **Poor** | **p** |
|  | n=358 | n=305 |  |
| Age, years | 69.8 ± 14.2 | 76.7 ± 12.6 | <0.0001 |
| Women, % | 41.1 | 50.5 | 0.015 |
| Latency time, min | 263.2 ± 164.3 | 310.4 ± 160.7 | <0.0001 |
| Previous modified Rankin scale | 0 [0, 1] | 1 [0, 1] | <0.0001 |
| Arterial hypertension, % | 63.7 | 68.5 | 0.218 |
| Diabetes, % | 23.5 | 23.3 | 0.519 |
| Smoking, % | 20.1 | 11.1 | 0.002 |
| Enolism, % | 14.2 | 12.8 | 0.649 |
| Dyslipemia, % | 39.1 | 35.7 | 0.377 |
| Peripherial arterial disease, % | 5.9 | 4.3 | 0.382 |
| Ischemic heart disease, % | 10.1 | 12.8 | 0.272 |
| Atrial fibrillation, % | 17 | 30.8 | <0.0001 |
| Previous TIA, % | 10.1 | 6.1 | 0.165 |
| Time since TIA < 1day, % | 6.7 | 3 | 0.077 |
| Axillary temperature at admission, ºC | 36.2 ± 0.6 | 36.7 ± 0.8 | <0.0001 |
| Basal glycaemia, mg/dL | 135.2 ± 55.4 | 142.2 ± 56.9 | 0.662 |
| Leukocytes, x10^3^/mL | 8.8 ± 2.9 | 9.8 ± 3.7 | 0.007 |
| Fibrinogen, mg/dL | 441.8 ± 108.2 | 462.7 ± 105.6 | 0.075 |
| C reactive protein, mg/L | 2.9 ± 3.6 | 4.7 ± 4.7 | <0.0001 |
| Glycosylated hemoglobin, % | 6.0 ± 1.1 | 6.0 ± 1.3 | 0.649 |
| LDL-cholesterol, mg/dL | 106.5 ± 33.9 | 109.5 ± 35.4 | 0.454 |
| HDL-cholesterol, mg/dL | 42.8 ± 17.7 | 42.6 ± 15.4 | 0.51 |
| Triglycerides, mg/dL | 111.1 ± 65.5 | 108.5 ± 52.6 | 0.518 |
| Sedimentation rate, mm | 24.5 ± 18.2 | 31.6 ± 26.1 | 0.004 |
| NT-proBNP, pg/mL | 1194.9 ± 1597.4 | 2865.3 ± 2778.3 | <0.0001 |
| Any Reperfusion treatment, % | 40.2 | 15.7 | <0.0001 |
| DWI volume at admission, mL | 20.5 ± 38.6 | 54.6 ± 74.7 | <0.0001 |
| Hemorrhagic transformation, % | 8.7 | 17.9 | <0.0001 |
| NIHSS at admission | 13 [7, 18] | 17 [13, 21] | <0.0001 |
| Infarct growh, % | 19.9 ± 29.4 | 30.1 ± 27.8 | <0.0001 |
| **TOAST** |  |  | <0.0001 |
| Atherothrombotic, % | 22.8 | 25 |  |
| Cardioembolic, % | 26.6 | 50.4 |  |
| Lacunar, % | 18.3 | 0.4 |  |
| Indeterminate, % | 32.4 | 24.2 |  |
| **Neurological Instability** |  |  | <0.0001 |
| Neutral | 39.4 | 34.4 |  |
| Positive | 58.4 | 37.7 |  |
| Negative | 2.2 | 27.9 |  |
| Glutamate (µM) | 41.031 ± 58.16 | 83.43 ± 115.68 | <0.0001 |
| IL6 (pg/mL) | 11.152 ± 5.79 | 11.031 ± 9.52 | 0.842 |

**Table S3.** Clinical and analytical variables for ischemic stroke patients classified in three groups according the neurological instability (neutral, positive and negative).

| **Neurological Instability** | | | | |
| --- | --- | --- | --- | --- |
|  | **Neutral** | **Positive** | **Negative** | **p** |
|  | n=246 | n=324 | n=93 |  |
| Age, years | 71.4 ± 13.8 | 73.6 ± 14.3 | 74.4 ± 12.5 | 0.069 |
| Women, % | 41.1 | 50.5 | 0.015 | 0.21 |
| Latency time, min | 343.9 ± 169.9 | 261.6 ± 178.4 | 339.2 ± 94.7 | <0.0001 |
| Previous modified Rankin scale | 0 [0, 1] | 0 [0, 1] | 1 [0, 1] | 0.048 |
| Arterial hypertension, % | 58.5 | 68.5 | 76.3 | 0.003 |
| Diabetes, % | 23.2 | 23.8 | 23.7 | 0.986 |
| Smoking, % | 19.5 | 14.8 | 10.8 | 0.105 |
| Enolism, % | 15.9 | 11.4 | 15.1 | 0.28 |
| Dyslipidemia, % | 36.6 | 38.9 | 35.5 | 0.773 |
| Peripherial arterial disease, % | 3.7 | 5.9 | 6.5 | 0.409 |
| Ischemic heart disease, % | 10.6 | 10.8 | 15.1 | 0.468 |
| Atrial fibrillation, % | 19.9 | 23.1 | 33.3 | 0.033 |
| Previous TIA, % | 8.1 | 8.6 | 9.7 | 0.902 |
| Time since TIA < 1day, % | 2.4 | 7.4 | 3.2 | 0.007 |
| Axillary temperature at admission, ºC | 36.3 ± 0.7 | 36.3 ± 0.6 | 37.0 ± 1.0 | <0.0001 |
| Basal glycaemia, mg/dL | 127.7 ± 53.7 | 129.9 ± 46.4 | 146.4 ± 63.3 | 0.49 |
| Leukocytes, x10^3^/mL | 8.7 ± 3.1 | 8.9 ± 2.8 | 10.3 ± 3.7 | 0.105 |
| Fibrinogen, mg/dL | 445.1 ± 113.8 | 459.3 ± 108.1 | 429.3 ± 105.8 | 0.402 |
| C reactive protein, mg/L | 2.8 ± 3.9 | 3.1 ± 3.0 | 5.2 ± 5.7 | 0.005 |
| Glycosylated hemoglobin, % | 6.0 ± 1.2 | 5.9 ± 0.9 | 6.1 ± 1.5 | 0.248 |
| LDL-cholesterol, mg/dL | 112.5 ± 36.3 | 106.3 ± 32.3 | 115.2 ± 46.1 | 0.144 |
| HDL-cholesterol, mg/dL | 42.0 ± 15.8 | 42.8 ± 14.9 | 43.8 ± 22.2 | 0.625 |
| Triglycerides, mg/dL | 107.2 ± 38.2 | 101.9 ± 41.2 | 106.3 ± 57.6 | 0.124 |
| Sedimentation rate, mm | 28.1 ± 23.5 | 26.5 ± 20.9 | 31.6 ± 27.9 | 0.05 |
| NT-proBNP, pg/mL | 1474.1 ± 1863.7 | 2201.6 ± 2333.1 | 2606.3 ± 2996.6 | 0.081 |
| Any reperfusion treatment, % | 22.4 | 69.8 | 7.8 | <0.0001 |
| **TOAST** |  |  |  | <0.0001 |
| Atherothrombotic, % | 15.4 | 25.6 | 28.0 |  |
| Cardioembolic, % | 33.7 | 39.2 | 48.4 |  |
| Lacunar, % | 26.4 | 1.9 | 1.1 |  |
| Indeterminate, % | 24.4 | 33.3 | 22.6 |  |
| Glutamate (µM) | 46.42 ± 84.34 | 53.31 ± 71.58 | 124.97 ± 138.20 | <0.0001 |
| IL6 (pg/mL) | 12.00 ± 8.31 | 8.98 ± 4.31 | 16.29 ± 11.79 | <0.0001 |

|  |  | **β** | **CI 95%** | **p** |  | **β *** | **CI 95%** | **p** |
| --- | --- | --- | --- | --- | --- | --- | --- | --- |
| **Glutamate (µM)** | | -0.027 | -0.042,-0.012 | <0.0001 |  | -0.029 | -0.046,-0.012 | 0.001 |
| **IL6 (pg/mL)** |  | -0.818 | -0.989,-0.648 | <0.0001 |  | -0.424 | -0.629,-0.219 | <0.0001 |
| **Latency time (min)** | | -0.044 | -0.053,-0.035 | <0.0001 |  | -0.034 | -0.044,-0.023 | <0.0001 |
| **Any reperfusion treatment** | | 10.296 | 7.002-13.588 | <0.0001 |  | 5.96 | 2.454,9.465 | <0.0001 |

**Table S4.** Multivariate linear regression model for neurological instability.

*Adjusted by previous mRS, time since last TIA less than 1 day, C-reactive protein and temperature.

**Figure S1** Clinical Markers predicting poor and good outcome at 3 months expressed as unadjusted Odds Ratios (95% confident interval).


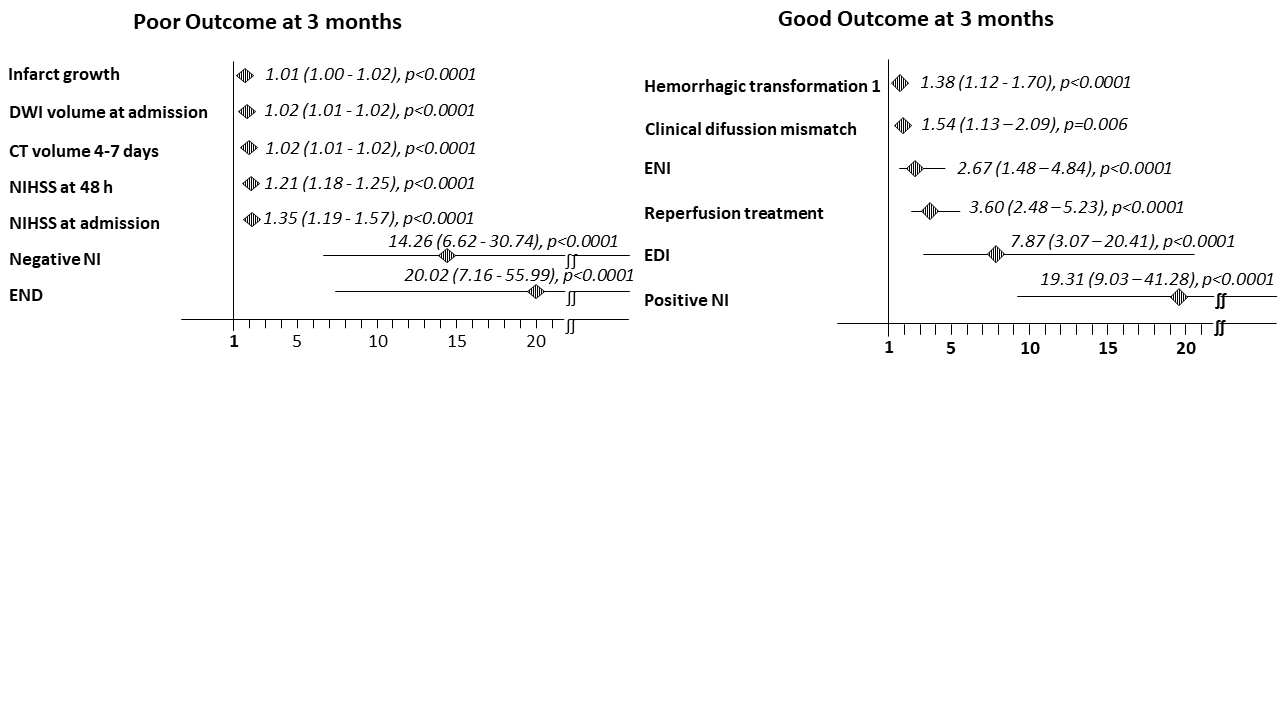


DWI: diffusion weighted image, CT: computer tomography, NIHSS: National Institute of Health stroke scale, NI: Neurological instability, END: early neurological deterioration, ENI: early neurological improvement, EDI: early dramatic improvement.

**Figure S2** Percentage of patients with NI calculated from the variations in the NIHSS between 0-48h; 0-24h and 24-48 h as a function of the latency time.
